# Low self–esteem in women with eating disorders and alcohol abuse as a psycho–social factor to be included in their psychotherapeutic approach

**Published:** 2010-11-25

**Authors:** G Iorgulescu

**Affiliations:** ‘Carol Davila’ University of Medicine and Pharmacy, Bucharest Romania

**Keywords:** eating disorders, psycho–sexual identity, alcoholism, gender/role atributes, masculinity/feminity index

## Abstract

Author have analyzed the psycho–social peculiarities of the women from Romania who are affected by eating disorders and alcohol excessive consumption, and studied the manner of the link between these disease and the psycho–sexual. 120 participants at the study (Oltenia district) were divided into 2 groups: 60 healthy women, 30 with eating disorders and 30 alcohol dependent women. In all subjects were applied the following tests: Scale for compulsive appetite (SCA) and Scale of interest for own weight, both for eating disorders, CAGE questionnaire for alcohol dependence and two scales for determining: the gender–role ambivalence (O'Neil and Caroll Scale) and the masculinity and feminity index (A. Chelcea). The results obtained in both lots of Romanian women with pathologic behavior (food and/or alcohol consumption) have indicated a low psycho–sexual identity versus control group but no correlation with masculinity/feminity index.

## Introduction

The studies made in the last decades gathered information about feeding disturbances and substances abuse. The researchers had examined personality traits, family history biological and cultural background. Due to the fact that a large number of persons with feeding problems are women (90–95%) must be considered the role of gender–masculinity and feminity–in the development of feeding problems and alcohol consumption.

It was suggested that both food and alcohol consumption disturbances are **manifestations of an addictive type of personality**. Other explanations include a) existence of a biological determination b) a dysfunctional family environment c) a fundamental struggle with a weak self control  d) insufficient abilities to cope with the stress and depression. However all these explanation ignores some other aspects of social and cultural context in which food and alcohol intake problems occur.

The study of psychosexual identity as well as the womanhood and manhood coefficient is another way to examine this context

In the same time undertaking a psychosexual identity is tight related to self acceptance, a component of the self with a wider conceptual sphere including other personality attributes: positives (qualities) and negatives (flaws).

Women with gender–role attributes feebly defined are much more probable to have a slender self acceptance thus making them more vulnerable to problems involving feeding and alcohol consumption.

Defining **self acceptance/ self esteem **(two notions not entirely overlapping) Crisp and Turner (2008) deemed that being an evaluative component of the self designated to establish our rank among the others including towards the standards / role models deemed being positive or negative. Self acceptance nuances the evaluation of self image (bringing into light of conscience of own thoughts, emotions, behaviors)  in axiological way , representing also an attitude of being or not in agreement with that image.

**Self acceptance** it makes reference to the adaptive role of self and it is expressed trough its continuous tendency to diminish the tension therefore ensuring the equilibrium and peace. Self acceptance has as main goal to gain the gratification on imaginative or symbolic level. Self acceptance ponder itself in a good positive opinion being compatible with a person full of trust and worth, a person who take both praises and disapprovals in an objective manner. Attitude of a positive self image person it is based on inner values and who accepts the consequences occurring from own behaviors. 

A low degree of self acceptance is translated in a negative opinion towards self and the conviction that others too have the same opinion.

That independent variable could have following levels: a low degree of self acceptance, a medium or normal degree, and a high degree of self acceptance.

Self acceptance plays a key role in development of a personality and a self and it's a major component of our emotional health

If both Kahn and Singha had discovered a positive correlation between low self esteem and feeding and alcohol intake disorders, then   Striegel–Moore (1993) opinionate that   stress induced by the image of own body could too determinates women to involve in abusive behaviors like excess drinking or  food disorders (bulimia anorexia obesity) . In other words, low self esteem could be both result and cause of such behaviors. The present work has as intention to analyze these personality traits from perspective of considering personality as a crucial factor in genesis of feeding disorders and alcoholism. 

## Theoretical Objectives

1. Discovering the influence of the social cultural environment in the rate of feeding disorders at women and more specific if under influence of social and cultural environment some people are more susceptible than others to feeding disorders and alcoholism. The purpose of this work is to see if in our country also the cultural pressure could have a negative effect on self esteem thus initiating and maintaining feeding and substance abuse problems.
2. Determining the relation between self acceptance and  problem related to food and alcohol problems.

## Practical Objectives

1. If we determine that women with alcohol and food intake problems are influenced by cultural pressure exerted on them and if we also determine the measure and the way in which such pressures are manifested would be easier to prevent and treat such problems.
2. The main practical objective of this work is related to fact that once we demonstrated the relation between alcohol and food abuse problems at women and social cultural influences involved in self esteem we could develop new programs of prevention and treatment

### Motivation

Why feeding related disorders at women?We choose the study of feeding related disorders because in our country also the number of people affected by problems like obesity, bulimia, strict and severe diets is in a continuous rising and studies upon Romanian population are rather scarce and the great majority the population affected by over mentioned problems are women.

Why alcoholism at women?The motivation of choosing studying alcoholism at women holds with the neglecting of this problem in the past. Because the number of persons affected by alcoholism is higher among men the problem of women's alcoholism was not enough studied and maybe therefore the number of women involved in alcohol related problem is raising in real life.

Therewith prevention and treatment programs regarding alcoholism in our country and not only, doesn't take account of clinical, psychological and social features specific to women with an alcohol related problem and thus the healing is more difficult to attain.

This study is motivated by the willing of a differentiate approach to alcoholism at women, and that approach should be regarded in the wider context of alcoholic woman's psychology 

## Research Hypothesis

General hypothesis: We suppose that women who manifest feeding and alcohol consumption problems will be low in self acceptance

Independent variable: disorders of feeding and alcohol consumption (TAA)

Dependent Variable: self acceptance In a future work we will analyze the link between eating and alcohol problems and psychosexual identity undertaking considering some other dependent variables such as gender role ambivalence, femininity and masculinity quotient.

## Samples

In the present experiment 120 women participated, 60 of them from general population witness group, and 60 women with alcohol and eating problems of which 30 with eating problems such as bulimia, obesity and harsh diets and 30 women with alcohol problems. Owe to large number of items and investigations required the size of work group is so small

All subjects of this experiment are from urban environment, more precisely from Bucharest and Ramnicu Valcea

Regarding the work group, subjects with eating and alcohol problems they came from municipal hospital Ramnicu Valcea, section 2 psychiatry (Dr. Creangă Silvia) and clinical hospital  Colentina (Prof. I.B. Iamandescu). They were tested between 23–29 12 2006 and 4–15 02 2007. 

Control group subjects without eating or alcohol problems were pupils and teachers from several high schools: Economic National College ‘Alexandru Lahovari’, National College ‘Mircea cel Batran’, and also students from UMF–University of Medicine and Pharmacy–dentistry section.

All of the 120 participant subjects are feminine gender with ages between 18 and 45, Romanian citizenship, Romanian nationals, orthodox confession, permanent inhabitants in those two cities Bucharest and Ramnicu Valcea.

Subjects were tested each Friday of the month between 10 and 13 AM being administrated all the tests consequently.

What about subjects in work group they were called by their psychiatry medic ant they were explained about the participation at a study and the answers done at the papers and questionnaires have no relevance and consequences at all upon their cure, or hospital situation and also there are no good or bad answers at all and no time limit.

All participant subjects, including hospital patients were quite cooperative 

## Apparatus (Materials, Scales, Instruments)

What about the apparatus used in the present experiment we used as materials: paper, pencil, (instruments and answering sheet) as well as chair and desk used during tests by subjects to respond to tests items.

Regarding the instruments of psychological testing that we used those were: compulsive appetite scale, Scale regarding diet and own weight preoccupation and Cage questionnaire, Scale of self acceptance.

1. Compulsive appetite Scale: it is a scale with eight items (each one having five levels) which measures the compulsive appetite associated with obesity (Dona M. Kagan and Rose L. Squires) with the purpose to measure uncontrollable appetite. The scores represent the sum of items values and may vary from 8 to 40. High scores reveals a higher compulsivity in somebody appetite.
2. Scale regarding diet and own weight preoccupation: made by Dona Kagan and Rose Squires it consist of 14 items designated to measure the interest for own weight and diet as result of a feeding disturbance. The main purpose of this scale: to measure dieting behaviour. At this scale the score represents the sum of values of all items and may vary from 14 to 70. High scores reflects a higher interest in dieting and maintaining own weight in certain limits.
3. Cage Questionnaire:  elaborated by R. Brown and L. Rounds and translated and adapted in Romanian by Elena Simionescu. This questionnaire was designated to test alcoholism and represents an acronym of four behavioral parameters at persons with drinking problems: Cut down–persistent wish to cut down alcohol consumption, Annoyed–continuous reproaches from those around him, Guilty–self culpabilisation, Eye opener–the morning shot as an invigorating treat
4. Scale of self acceptance created by Emanuel Berger one of the oldest scales that measures the self acceptance and self esteem. Description: The inner image of the self, present in everybody's mind is a portrait based on our own social experiences. Based upon earlier activity of Dr. Elisabeth Scheerer and Dr. Carl Rogers, Dr. Berger listed 9 characteristics of self acceptance such as: ‘He have trust in his own capabilities to manage his way in life’. This formula lead to selection and development of a final group of 36 items for self, used to build the self acceptance scale. That scale is frequently used today in the whole world as well as for research and also in medical psychology and psychiatry to measure and characterize in a multi axial way the self esteem and self acceptance.

**Low scores (0–110) **express a **low self acceptance**. Such a score could be translated as a negative image on own self and the feeling that the others have the same bad image on that person. 

**Medium scores (111–150) **reveals a normal level of self acceptance; however that means ups and downs could occur from a day to another depending on the specific of the situation and therefore a better or less better impression on own self appears on daily basis.

**High scores (151–180)** show us how that person is full of confidence and trust in his/her own worth. The others may see that person being very affable because he/she accepts both praises and critics in an objective manner. Attitude of such a person is based upon inner values and accepting consequences resulting from own behaviors. 

## Methods|materials

The present research has the purpose to demonstrate the existence of a correlation between alcohol and eating disorders (TAA) on a side and self acceptance on another, following that in other work to be analyzed the results of correlation between psychosexual identity and masculinity and femininity quotient.

Independent variable is constituted by the problems related to eating and drinking. About eating we have in view women   diagnosed with bulimia, anorexia, obesity and harsh, punishing diets. What about drinking problems, in view were women with dependency and frequent abuse of alcohol. 

In all hypotheses independent variable is the same: the women with feeding and drinking problems. Instead the dependent variable takes different values.

In the first hypothesis regarding correlation between eating disorders and self acceptance the self acceptance is the dependent variable.

To testing hypotheses we gathered three experimental groups. 

Experimental group 1 witness group composed by the 60 women without drinking and eating disorders. Experimental group 2 work group 1 composed by those 30 women with eating disorders. Experimental group 3 work group 2 composed by the rest of 30 women diagnosed with drinking problems

In the first general hypothesis the result obtained at self acceptance test by control group and work group 1 were compared. Because admittance in those two work groups was made upon positive results at those two scales regarding eating problems (1. compulsive appetite 2. diet and weight control preoccupation) on a side and CAGE questionnaire regarding overdrinking on the other side, only a comparative display of the results obtained at the Berger scale of self acceptance by both groups will be made.

## Results

The result were corroborated with psychiatrist   diagnoses  and they are displayed at Table 1 and Table 2

General hypothesis 1: If women present eating disorders then they will have a lower self acceptance. To verify this we use the self acceptance scale applied to all subjects. The results may vary from 0 pts. to 180 pts. 

The results of the subjects entered in the following ranges of values**Control group –95–143; Work groups 1, 2–59–126**

To test the hypothesis we use **t test**–student test–for independent two–sample

**Figure 1:**
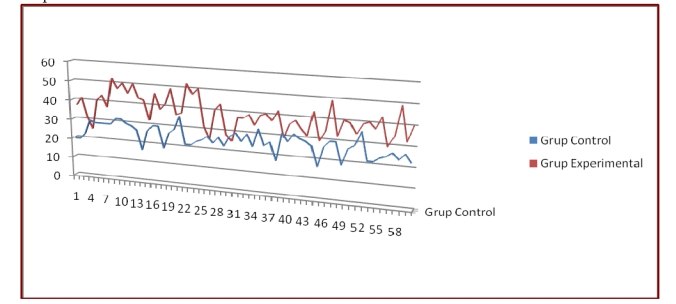
Results

The control group was the first sample and the two workgroups put together were the second sample. Test of self acceptance were applied to both samples ant the results are shown in Figure 1

In order that **Student's t test** to be applied we need a normal distribution, therefore indices of **skewness** and **kurtosis**, were calculated. The value of those two indices must be between–1+1 as a condition for the test to be applied. The result being in the range the test could be applied on those independent two samples.  

As a result of t test applied on the two independent samples (t =7,992) we obtained a significance threshold equal to 0,007. The value of this threshold is very important to determine some correlations between the two variables from hypothesis. Our threshold value of 0,007 < 0,05 indicates that is a relation between the variables.

**Table 1:** Results A

	Control Group	Experimental group 1 w. eating disorders	Experimental group 2 w. alcohol disorders
No. women participating	60	30	30
No. persons with results btw.: 0–110	22	30	25
No. persons with results btw: 111–150	38	0	5
No.persons with results btw.: 151–180	0	0	0

Self evaluating scale regarding self esteem and acceptance reveals values significantly lower at the two work groups respectively with feeding and alcohol problems. Comparsion of the mean values of the studied variable revealed results significantly different

**Table 2:** Results B

control group	N=60	Experimental Group 1,2	N=60
m=117,37		m=91,95	

**Mean value of self acceptance quotient is significantly higher in control group (m=117,37) than the mean value obtained in those two work groups put together (m=91,95)**. It is obvious that first value, obtained in control group it is situated in the range of medium scores (111–150) what means a positive self image and self acceptance, while the second mean is situated in the range of low scores that signify a poor self image and problems with self acceptance.

## Discussions

1. The present research gives us the opportunity to collate a sample constituted by women without eating and drinking problems–control group–with a sample made by women with eating disturbances to see if **there are significant differences between those two groups regarding self acceptance**. Data obtained by applying statistical tests indicate the existence of a significant difference toward self acceptance
2. To determine the levels of self acceptance we had applied the Scale of self acceptance to all subjects of this research. The starting point being the hypothesis of lower self esteem at women with eating disorders
3. Analyzing the results presented earlier we can see that the great number of problems free women are situated in the medium range value of self acceptance quotient while a great number of women who have feeding and alcohol problems are situated in the low range of self esteem quotient.
4. Previous researches–Krahn and Singha, as well as  Striegel Moore –testify the presence of a negative self image to women with eating problems who use adjectives like ‘grotesque’, ‘fat’  or ‘disgusting’ and frequently bodily image have no correspondence in reality, hereby their body weight being in normal limits sees herself as being massively overweight
5. Also to a great number of TAA women that we studied  there were noticed affective symptoms including dysphoria, self depreciation, loss of focus, lack of interest, hopelessness, sleep disorders, suicidal thoughts. Those aspects support the connection between affective and eating disorders
6. Importance of studying the self image, lowered at women with TAA–according to our study–resides in fact that the psychologist solicited to treat the addictive behaviors of those women patients,  finds an highly effective action point to  begin with his psychotherapeutic and also situational  actions designated to cut off the vicious circle like TAA–negative self image–TAA
7. The psychotherapist should investigate the causes–others than TAA effect–meant to affect AS at women under psychological or psychopharmacology interventions.
  The psychoterapist should know the TAA pacient's personality and her biografic context, first of all the relations between the pacient and the ones from ‘ecological niche’ (Willi) in which they exist, meaning the relationships with the close ones (cuples, famility, colleagues).
  Besides psycho–social factors annihilation, the psychoterapist should take into consideration another variables from social psychology domain, capable of recontruct self–esteem to any individual–this is also applicable in women with low TAA and AS.
  We should have in mind that self–esteem it's not something rigid, inextensibil and incapable of changes–it can vary depending on life changing situations (Crisp and Tuner).
8. There were several cases in which the cuple's relationship might have influenced the TAA etiopatogenia.
  The persistency of these kind of relation could be the starting point for a low AS or the cause of TAA–these could be triggered by afective trauma, depression.
  Another vicious circle closes when the ‘prosecutor’ (the partner) is accusing the guilty pair–caused trap addictive behavior–the effects of nutritional imbalance, manifested in the physical aspects or the negative social consequences of altered relationships with entourage.
  This kind of partner could encourage (especially in alchoolism) the practice of this fault, as long as excesive eating, but then could became critical accusing the partner (while having already low AS)–unsightly the concequences–these could cause breaking the couple and then to a even lower AS of the pacient.
  The psychotherapist, trained to deal with this kind of situations could try to remove the pacient from the influence of non–coresponding partner or could try to build and improve the relationship between themselves, especially when talking about married couples.
9. Compensating low self–esteem could be done through incresing, on psychoterapyst advice, the social interaction of TAA pacients (these is applicable for the ones already under recovery program), as per Steele theory (‘self affirmation therapy’) who considers that when the self–esteem is low or threaten, the individual will have a compensatory behavior, due to reveal some real qualities, ‘un–exposed’ before.
10. Most of the pacients with low self–esteem tend to isolate and we must fight against this aspect. TAA consequenses (phisical and behavioral) tends to underline the tendancy to social retraction.
  To the satisfaction of each person's need for affiliation (Linton), the concept ‘**including other in the self**’ (Aron et al.–1992) it refers to the human tendency to share with their loved ones everything that feels good–usual reserved for themselfes.
  The psychoterapyst could exploit in favor of low AS pacients this tendancy, encouraging affective investment towards people (especially children and trusted friends), pets etc;, having the real chance for the studied pacients to recover their low AS and heal TAA (of course adopting also others terapeutical measures).
11. On the other side, on virtue of the social identity of an individual, tends to maintain himself within the membership group even though in here he has a lower status which can generate a low self–esteem.
  Raising self–esteem and self acceptance could be done spontanously (but not always) in two ways:
  - Social affirmation
  - Dissociation–from personal traits, group specific–of those which are non–essential, which he has more than the rest of the group (‘Disidentity’–Tajfel and Tuner)
  It is not sure of the TAA women and with low self–esteem use these spontaneous mechanism of resoving the self–esteem gap between the group members, but it is sure that a benefic role cand be played both by satisfaying the need of group affiliation and by the possibility of  adjusting the self–esteem through the affiliation.
  Therefore, taking into consideration the above evidence, the psychoterapyst can insist on the necesity of TAA women with low self–esteem to use not only spontaneously but also ‘controled’ by the therapist of those possibilities to use social contacts. We can speak about an ‘genuine therapeutic plot’ capable of raising self–esteem and correcting the addictive behavior (Sedikides and Gregg–2002).
12. Must not be omitted the fact that not all the persons with eating and weight problems are accompanied by a low self esteem and a negative self image, especially when those trait are moderate and coexist with  immunogenic traits of personality such as optimism, robustness well and thoroughly expressed (‘Jovials obesity’ – Iamandescu).

## Synopsys of conclusions 

Previous researches as well as our study proves that it is a strong connection between the eating and drinking disorders–TAA–studied together  and self acceptance. What is less known is in what measure the negative self image and a low self esteem are causes or effects of feeding and drinking problems. Further more there are following important aspects clarify: existence and type of correlations between various affective disorders and eating and drinking disorders and also those correlations in the specific social cultural environment and conditions of Romania.

In the frame of this study we have had in view to track down the psychological and social specificity of alcoholism and eating related disorders at women from Romania. 

The present study has as aim to demonstrate the relation between self acceptance and self image and eating and drinking related disorders at 120 subjects. The allotment was as following: a work group of 60 women, 30 of them with eating disorders, 30 with alcohol related problems, an witness group of 60 women from the rest of population without problems related to eating and drinking.

The tests applied to diagnose TAA were the following: ‘compulsive appetite scale’, ‘scale regarding diets and own weight preoccupation’, ‘Cage questionnaire’ about drinking problems and ‘Berger scale of self acceptance’

The results of first three scales were collated to the result of the last scale ‘Berger’ applied to all 80 subjects.

Following analysis and data interpretation results that low self acceptance and a negative self image are characteristics of  women with TAA alcohol and eating disorders

The conclusions of the present study offers to the psychologist who is required to solve such a problems an effective and efficient starting point in breaking the vicious circle constitute by TAA–negative self image –TAA
